# The joint effects of sleep duration and exercise habit on all-cause mortality among Chinese older adult: a national community-based cohort study

**DOI:** 10.3389/fpubh.2025.1538513

**Published:** 2025-03-26

**Authors:** Na Li, Kexin Ren, Yuan Tao

**Affiliations:** ^1^College of Physical Education, Jilin Normal University, Siping, Jilin, China; ^2^College of Mathematics and Computer, Jilin Normal University, Siping, Jilin, China

**Keywords:** sleep duration, exercise habit, all-cause mortality, Chinese older adult, CLHLS

## Abstract

**Abstract:**

This study examines the combined effects of sleep duration and exercise habits on all-cause mortality in older Chinese adults using data from the Chinese Longitudinal Healthy Longevity Survey (CLHLS).

**Methods:**

Data were collected from 7231 residents aged 60 and above from the CLHLS. Participants were categorized based on their sleep duration (short sleep: <6 hours, normal sleep: 6–8 hours, and long sleep: >8 hours) and exercise habits (physically active, physically inactive, inactive-to-active, and active-to-inactive). The analysis was conducted over three follow-up periods (2011, 2014, and 2018). Cox proportional hazards regression models were used to assess the associations between sleep duration, exercise habits, and all-cause mortality.

**Results:**

The results showed that compared to short sleepers, individuals with normal sleep (6–8 hours) had a slightly reduced risk of death, although this reduction was not statistically significant (HR = 0.97, 95% CI 0.87–1.08). In contrast, long sleepers (>8 hours) had a significantly higher risk of mortality (HR = 1.28, 95% CI 1.16–1.43). Regarding exercise habits, regular exercise or transitioning from inactivity to exercise significantly reduced mortality risk compared to those who did not exercise (HR = 0.74, 95% CI 0.66–0.83 and HR = 0.88, 95% CI 0.80–0.97, respectively). Notably, exercise habits did not significantly impact mortality among short sleepers, regardless of gender. However, both men and women with normal or long sleep experienced significant mortality benefits from regular exercise. Additionally, older women who transitioned from a sedentary lifestyle to physical activity during periods of long sleep demonstrated lower mortality rates.

**Conclusion:**

Both sleep duration and exercise habits are associated with mortality risk among older Chinese adults, with notable gender differences in their combined effects. Targeted health policies that encourage improved sleep and exercise habits, while considering gender-specific needs, are essential to reduce mortality and enhance quality of life in this population.

## 1 Introduction

Sleep duration and exercise habits are critical lifestyle factors that significantly influence health outcomes, particularly among the older adult population. As modifiable lifestyle factors, both sleep and physical activity have been associated with improved health and longevity (1, 2). With the rapid increase in the older adult population in China (3), understanding how these two factors impact health within this demographic is of utmost importance.

While previous studies have thoroughly examined the individual effects of sleep and exercise on health outcomes, the combined effects of these factors remain understudied, particularly in the context of the Chinese older adult population. The Seniors-ENRICA cohort study in Spain demonstrated that appropriate physical activity mitigates the impact of poor sleep duration on mortality among older adults (4). Similarly, the UK Biobank study found that physical activity and sleep duration interacted with the risk of all-cause mortality, as measured by accelerometers (5). In contrast, a Brazilian study indicated no significant association between resting activity rhythms and mortality when physical activity was considered. This suggests that regular exercise may drive the resting activity rhythm pattern, making physical activity a key influencing factor in this relationship (6). Notably, only one relevant study involving Chinese older adults (7) has investigated the association between sleep scores, leisure-time physical activity, and all-cause mortality. While this study emphasized the potential benefits of improved sleep and increased physical activity in reducing mortality, it should be noted that it was conducted on retired employees of the Dongfeng-Tongji (DFTJ) Group and did not represent a nationwide sample. These findings suggest that physical activity and sleep may interact through different mechanisms, affecting the health and longevity of older adults. Therefore, further research is needed to deepen our understanding of the joint effects of these factors across diverse populations, particularly among the Chinese older adult.

The imperative to study the health behaviors and outcomes among Chinese older adult is underscored by its rapid demographic expansion, a trend driven by increased life expectancies and declining birth rates (8). This substantial demographic shift not only poses challenges to public health and healthcare systems but also necessitates tailored approaches to health promotion and disease prevention. The unique cultural practices and health beliefs within this population may significantly influence their sleep and exercise habits, which are critical factors in determining health outcomes (9). Understanding these nuances is essential for developing effective and culturally sensitive interventions to support the health and well-being of Chinese older adult.

The aim of this study is to investigate the joint effects of sleep duration and exercise habits on all-cause mortality in older adults in China, along with the associated mechanisms of action. This study will employ a national community-based cohort design and will be the first to examine how changes in exercise habits and sleep duration impact all-cause mortality. The findings will provide valuable insights into the most effective combinations of sleep and exercise. Additionally, these results may inform public health interventions targeting the older adult population in China and significantly contribute to our understanding of how sleep and exercise can be leveraged to improve health outcomes in this demographic.

## 2 Methods

### 2.1 Data sources

The data for this study are derived from the Chinese Longitudinal Healthy Longevity Survey (CLHLS), which is the largest cohort study focusing on the older adult population in China. This study was organized by the Centre for Healthy Ageing and Development at Peking University in collaboration with the National Institute for Development Research. The CLHLS encompasses 23 provinces, municipalities, and autonomous regions, with a total of 113,000 household interviews conducted. Approximately half of the cities and counties in 22 of the research provinces (excluding Hainan Province) were randomly selected as research sites. The survey received approval from the Institutional Review Board of Peking University (IRB00001052-13074), and all participants, or their legal representatives, provided written informed consent.

We utilized a sample of individuals aged 60 and older from the 2011 follow-up study, and all participants were evaluated again in 2014 and 2018, with ongoing monitoring until death, loss to follow-up, or the study’s end. Participants with incomplete records were excluded from the analysis. The final sample size for analysis is 7,231 (refer to Supplementary Figure 1).

### 2.2 Variable measurement

#### 2.2.1 Exposure

In the survey, participants were asked, ‘Do you exercise frequently at present?’ and ‘Did you exercise frequently in the past?’ Based on their answers to these two questions, participants were classified into four groups: (1) physically active if they exercised frequently both in the past and now, (2) physically inactive if they did not exercise frequently either in the past or the present, (3) inactive-to-active if they did not exercise frequently in the past but are doing so now, and (4) active-to-inactive if they exercised frequently in the past but are not currently active (10).

Sleep duration (in hours) was defined as the amount of sleep reported by the participant in the self-reported questionnaire. The exact amount of time was based on the following question: ‘How many hours of sleep do you currently get on a typical day?’ Using restricted cubic spline curves and threshold effect analyses (11), we determined that the threshold of sleep time associated with all-cause mortality was 8 h (Supplementary Figure 2). Based on the threshold effects and classifications from previous studies (12, 13), we categorized sleep duration into three groups: short sleep (<6 h), normal sleep (6–8 h), and long sleep (>8 h).

#### 2.2.2 Outcome

The outcome of this study was all-cause mortality. Data on participant survival and date of death were collected during three rounds of follow-up in 2011, 2014, and 2018. Participants who remained alive or were lost to follow-up were assessed using the most recent point of contact (30 December 2018).

#### 2.2.3 Covariates

Based on previous studies of physical activity and mortality, as well as sleep duration and mortality, we included a variety of covariates that might influence the results: age (60–70, 70–80, >80), gender (male, female), marital (married, others), education years (continuous), smoke (yes, no) (14), drink (yes, no) (15), Self-assessed health (very bad, bad, fair, good, very good) (16), economic state (bad, good) (17), disease (no, yes) (18), body mass index (BMI) (continuous) (19).

### 2.3 Statistical analysis

The baseline characteristics of 7,231 participants were described based on sleep duration, with categorical variables expressed as frequencies and percentages, and quantitative variables expressed as means with standard deviations (SD). Comparison of baseline characteristics among sleep duration categories was performed using the *χ*^2^ test and one-way analysis of variance for categorical and continuous variables, respectively. We first analyzed the effect of sleep duration and exercise habits on all-cause mortality using multi-model cox regression: Model 1 without covariates, Model 2 with covariates including age, gender, education, and marital status, and Model 3 with covariates including age, gender, education, marital status, smoking, drinking, health status, economic state, disease and BMI. We then further analyzed the joint effect of sleep duration and exercise habits on all-cause mortality using stratified analyses by sex and interaction analyses. Data processing and analysis were performed using R version 4.3.0, along with the Storm Statistical Platform^1^. A two-tailed *p* < 0.05 was considered to be statistically significant.

## 3 Results

### 3.1 Basic characteristics

Table 1 shows the basic characteristics of participants based on their sleep duration. Among the total sample of 7,231 participants, there were 1,120 short sleepers (i.e., those sleeping less than 6 h), 3,743 normal sleepers (6–8 h), and 2,368 long sleepers (more than 8 h). Further analyses revealed that the proportion of both short and long sleepers increased among individuals aged over 80 compared to those with normal sleep duration. Moreover, the ratio of long sleepers to short sleepers was notably higher in the male group. In terms of marital status, individuals who were unmarried exhibited a significantly higher proportion of long sleepers compared to short sleepers. Similarly, non-smokers displayed a higher-than-average ratio of long to short sleepers. When examining drinking habits, drinkers showed a significantly higher proportion of long sleepers as well. The data suggest that short sleepers are more likely to have poor economic status, suffer from diseases, and be inactive compared to those with normal or long sleep durations. Additionally, it is noteworthy that individuals who reported a Self-assessed health of “good” or above had a significantly higher proportion of long sleepers. All observed differences are statistically significant (*p* < 0.05).

**Table 1:** Baseline characteristics of older adults stratified by sleep duration in CLHLS 2011–2018.

Variables	Total (*n* = 7,231)	Short sleep (*n* = 1,120)	Normal sleep (*n* = 3,743)	Long sleep (*n* = 2,368)	Statistic*	*p*
Age (years), *n*(%)					*χ*^2^ = 221.62	<0.001
60 ~ 70	578 (7.99)^#^	61 (5.45)	410 (10.95)	107 (4.52)		
70 ~ 80	2,124 (29.37)	338 (30.18)	1,257 (33.58)	529 (22.34)		
>80	4,529 (62.63)	721 (64.38)	2076 (55.46)	1732 (73.14)		
Gender, *n*(%)					*χ*^2^ = 42.28	<0.001
Female	3,371 (46.62)	426 (38.04)	1837 (49.08)	1,108 (46.79)		
Male	3,860 (53.38)	694 (61.96)	1906 (50.92)	1,260 (53.21)		
Marital status, *n*(%)					*χ*^2^ = 101.28	<0.001
Others	4,378 (60.54)	701 (62.59)	2067 (55.22)	1,610 (67.99)		
Married	2,853 (39.46)	419 (37.41)	1,676 (44.78)	758 (32.01)		
Education (years), Mean ± SD	2.49 ± 3.63	1.99 ± 3.17	2.86 ± 3.83	2.14 ± 3.42	*F* = 41.63	<0.001
Smoke, *n*(%)					*χ*^2^ = 9.60	0.008
No	5,806 (80.29)	929 (82.95)	2,958 (79.03)	1919 (81.04)		
Yes	1,425 (19.71)	191 (17.05)	785 (20.97)	449 (18.96)		
Drink, *n*(%)					*χ*^2^ = 7.70	0.021
No	5,884 (81.37)	943 (84.20)	3,039 (81.19)	1902 (80.32)		
Yes	1,347 (18.63)	177 (15.80)	704 (18.81)	466 (19.68)		
Health, *n*(%)					*χ*^2^ = 190.29	<0.001
Very bad	86 (1.19)	28 (2.50)	37 (0.99)	21 (0.89)		
Bad	1,121 (15.50)	297 (26.52)	509 (13.60)	315 (13.30)		
Fair	2,646 (36.59)	428 (38.21)	1,406 (37.56)	812 (34.29)		
Good	2,560 (35.40)	292 (26.07)	1,343 (35.88)	925 (39.06)		
Very good	818 (11.31)	75 (6.70)	448 (11.97)	295 (12.46)		
Economic state, *n*(%)					*χ*^2^ = 21.32	<0.001
Bad	5,942 (82.17)	968 (86.43)	3,079 (82.26)	1895 (80.03)		
Good	1,289 (17.83)	152 (13.57)	664 (17.74)	473 (19.97)		
Disease, *n*(%)					*χ*^2^ = 38.71	<0.001
No	2,733 (37.80)	345 (30.80)	1,401 (37.43)	987 (41.68)		
Yes	4,498 (62.20)	775 (69.20)	2,342 (62.57)	1,381 (58.32)		
BMI (kg/m^2^), Mean ± SD	22.08 ± 23.10	21.77 ± 9.71	22.51 ± 30.55	21.56 ± 10.44	*F* = 1.33	0.264
Exercise, *n*(%)					*χ*^2^ = 60.68	<0.001
Inactive	3,525 (48.75)	561 (50.09)	1803 (48.17)	1,161 (49.03)		
Active	1,189 (16.44)	137 (12.23)	730 (19.50)	322 (13.60)		
Inactive-to-active	1,649 (22.80)	284 (25.36)	795 (21.24)	570 (24.07)		
Active-to-inactive	868 (12.00)	138 (12.32)	415 (11.09)	315 (13.30)		

^#Percentage of categorical variables.^

*The *χ*^2^ test was used for categorical variables and analysis of variance for continuous variables.

BMI, body mass index; SD, standard deviation.

### 3.2 Multi-model cox regression analysis

In this study, we delved into the impacts of sleep duration and diverse exercise habits on all-cause mortality by employing multi-model cox regression analysis. Table 2 presents the findings from three distinct models (Model 1, Model 2, and Model 3), each incorporating various combinations of variables.

**Table 2:** Effect of sleep duration and different exercise habits on all-cause mortality in CLHLS 2011–2018.

Variables	Model 1		Model 2		Model 3	
	HR (95%CI)	*p*	HR (95%CI)	*p*	HR (95%CI)	*p*
Sleep duration						
Short sleep <6 h	1.00 (Reference)		1.00 (Reference)		1.00 (Reference)	
Normal sleep 6-8 h	0.80 (0.72 ~ 0.89)	**<0.001**	0.97 (0.87 ~ 1.08)	0.572	0.97 (0.87 ~ 1.08)	0.574
Long sleep >8 h	1.32 (1.19 ~ 1.47)	**<0.001**	1.28 (1.15 ~ 1.43)	**<0.001**	1.28 (1.16 ~ 1.43)	**<0.001**
Exercise						
Inactive	1.00 (Reference)		1.00 (Reference)		1.00 (Reference)	
Active	0.62 (0.56 ~ 0.70)	**<0.001**	0.73 (0.65 ~ 0.82)	**<0.001**	0.74 (0.66 ~ 0.83)	**<0.001**
Inactive to active	0.81 (0.74 ~ 0.88)	**<0.001**	0.88 (0.80 ~ 0.96)	**0.005**	0.88 (0.80 ~ 0.97)	**0.007**
Active to inactive	1.20 (1.08 ~ 1.33)	**<0.001**	1.09 (0.98 ~ 1.21)	0.101	1.10 (0.99 ~ 1.22)	0.072

HR, Hazard Ratio; CI, Confidence Interval.

Model 1: Crude.

Model 2: Adjust: age, gender, education, marital status.

Model 3: Adjust: age, gender, education, marital status, smoke, drink, health, economic state, disease, BMI.

Bold values indicate statistically significant (*p* < 0.01).

Initially, we discerned a notable influence of sleep duration on all-cause mortality. Specifically, individuals with long sleep duration exhibited a significantly elevated risk of mortality, with hazard ratios (HR) of 1.32 (95% CI 1.19–1.47) in Model 1, 1.28 (95% CI 1.15–1.43) in Model 2, and 1.28 (95% CI 1.16–1.43) in Model 3, all indicating a robust association. In contrast, those with normal sleep duration of 6–8 h showed a reduced risk of mortality compared to the reference group of short sleep duration in Model 1, with a HR of 0.80 (95% CI 0.72–0.89), but this association did not persist in the fully adjusted Model 3 (HR of 0.97, 95% CI 0.87–1.08).

Furthermore, our analysis unveiled a significant impact of exercise habits on all-cause mortality, indicating that being active leads to a significantly lower risk of mortality across all models. Specifically, the HR were 0.62 (95% CI 0.56–0.70) in Model 1, 0.73 (95% CI 0.65–0.82) in Model 2, and 0.74 (95% CI 0.66–0.83) in Model 3. Conversely, transitioning from an active to an inactive lifestyle was associated with a higher risk of mortality; however, this association was not statistically significant in the fully adjusted Model 3 (HR of 1.10, 95% CI 0.99–1.22). These findings underscore the importance of maintaining a healthy sleep duration and an active lifestyle to mitigate the risk of all-cause mortality.

### 3.3 Joint effects of sleep duration and exercise habits on all-cause mortality

To further investigate the combined influence of sleep duration and exercise habits on all-cause mortality, we conducted sex-stratified and interaction analyses.

#### 3.3.1 Older adult Chinese men

Table 3 provides a detailed breakdown of how these factors jointly affect all-cause mortality among older men. Among those with short sleep duration (<6 h), the (HR) for active individuals was 1.151 (95% CI 0.759–1.747) in the unadjusted model, which increased to 1.585 (95% CI 1.019–2.465) in the adjusted model, indicating a significant elevation in mortality risk (*p* = 0.041). Conversely, transitioning from an inactive to an active lifestyle was not significantly associated with mortality risk in either model. However, transitioning from an active to an inactive lifestyle showed a significant increase in mortality risk in both models, with HR of 1.538 (95% CI 1.024–2.312) and 1.555 (95% CI 1.023–2.363) respectively, highlighting the protective effect of maintaining an active lifestyle.

**Table 3:** The joint effects of exercise habits and sleep duration on all-cause mortality in older adult Chinese men.

Variables	Unadjusted model		Adjusted model*	
	HR (95% CI)	*p*	HR (95% CI)	*p*
Short sleep < 6 h (*N* = 426)				
Inactive	1.00(Reference)		1.00(Reference)	
Active	1.151 (0.759 ~ 1.747)	0.508	1.585 (1.019 ~ 2.465)	**0.041**
Inactive to active	1.024 (0.725 ~ 1.447)	0.893	1.152 (0.800 ~ 1.657)	0.447
Active to inactive	1.538 (1.024 ~ 2.312)	0.038	1.555 (1.023 ~ 2.363)	**0.039**
*P* for interaction	0.412		**0.032**	
Normal sleep 6–8 h (*N* = 1837)				
Inactive	1.00(Reference)		1.00(Reference)	
Active	0.695 (0.564 ~ 0.857)	**0.001**	0.733 (0.588 ~ 0.915)	**0.006**
Inactive to active	0.900 (0.737 ~ 1.098)	0.299	0.910 (0.743 ~ 1.115)	0.363
Active to inactive	1.327 (1.058 ~ 1.664)	**0.014**	0.969 (0.769 ~ 1.220)	0.787
*P* for interaction	**<0.001**		**<0.001**	
Long sleep > 8 h (*N* = 1,108)				
Inactive	1.00(Reference)		1.00(Reference)	
Active	0.755 (0.597 ~ 0.956)	**0.020**	0.742 (0.579 ~ 0.949)	**0.018**
Inactive to active	0.929 (0.758 ~ 1.138)	0.477	0.911 (0.742 ~ 1.119)	0.375
Active to inactive	1.065 (0.832 ~ 1.363)	0.616	0.997 (0.775 ~ 1.283)	0.981
*P* for interaction	0.265		0.803	

*Adjusted model to add covariates age, gender, education, marital, smoke, drink, health, economic state, disease BMI.

Bold values indicate statistically significant (*p* < 0.05).

For individuals with normal sleep duration (6–8 h), being active was associated with a significantly reduced risk of mortality in both models, with HRs of 0.695 (95% CI 0.564–0.857) and 0.733 (95% CI 0.588–0.915) respectively. The transition from inactive to active did not significantly alter the risk of mortality, while transitioning from active to inactive showed a significant increase in risk, with HR of 1.327 (95% CI 1.058–1.664) and 0.969 (95% CI 0.769–1.220) respectively, although the latter was not significant in the adjusted model.

Among those with long sleep duration (>8 h), the active lifestyle was associated with a reduced risk of mortality, with HR of 0.755 (95% CI 0.597–0.956) and 0.742 (95% CI 0.579–0.949) in the unadjusted and adjusted models, respectively. The transition from inactive to active did not significantly affect mortality risk, while transitioning from active to inactive showed a non-significant increase in risk.

The interaction terms were significant in the adjusted models for short and normal sleep durations, indicating that the effect of exercise habits on mortality risk varies depending on sleep duration.

#### 3.3.2 Older adult Chinese women

In contrast, Chinese older adult women exhibited a distinct pattern regarding the joint effects of sleep duration and exercise habits on all-cause mortality compared to their male counterparts, as illustrated in Table 4.

**Table 4:** The joint effects of physical activity and sleep duration on all-cause mortality in older adult Chinese women.

Variables	Unadjusted model		Adjusted model*	
	HR (95% CI)	*p*	HR (95% CI)	*p*
Short sleep < 6 h (*N* = 694)				
Inactive	1.00(Reference)		1.00(Reference)	
Active	0.654 (0.430 ~ 0.996)	**0.048**	0.816 (0.527 ~ 1.263)	0.361
Inactive to active	0.748 (0.561 ~ 0.997)	**0.048**	0.916 (0.681 ~ 1.232)	0.562
Active to inactive	1.096 (0.774 ~ 1.553)	0.606	1.158 (0.807 ~ 1.660)	0.425
*P* for interaction	0.972		0.762	
Normal sleep 6–8 h (*N* = 1906)				
Inactive	1.00(Reference)		1.00(Reference)	
Active	0.488 (0.379 ~ 0.628)	**<0.001**	0.701 (0.541 ~ 0.909)	**0.007**
Inactive to active	0.648 (0.528 ~ 0.794)	**<0.001**	0.879 (0.714 ~ 1.081)	0.221
Active to inactive	1.207 (0.968 ~ 1.506)	0.094	1.342 (1.074 ~ 1.677)	**0.010**
*P* for interaction	**<0.001**		**<0.001**	
Long sleep > 8 h (*N* = 1,260)				
Inactive	1.00(Reference)		1.00(Reference)	
Active	0.525 (0.381 ~ 0.725)	**<0.001**	0.561 (0.405 ~ 0.778)	**<0.001**
Inactive to active	0.721 (0.595 ~ 0.875)	**<0.001**	0.785 (0.646 ~ 0.955)	**0.015**
Active to inactive	1.088 (0.873 ~ 1.356)	0.454	1.088 (0.870 ~ 1.362)	0.460
*P* for interaction	0.961		0.268	

*Adjusted model to add covariates age, gender, education, marital, smoke, drink, health, economic state, disease BMI.

Bold values indicate statistically significant (*p* < 0.05).

Among those with short sleep duration (<6 h), active individuals exhibited a significantly reduced risk of mortality in the unadjusted model, with a HR of 0.654 (95% CI 0.430–0.996), which was not statistically significant in the adjusted model (HR 0.816, 95% CI 0.527–1.263). Transitioning from an inactive to an active lifestyle was associated with a significant reduction in mortality risk in the unadjusted model (HR 0.748, 95% CI 0.561–0.997), but this association was not significant in the adjusted model. Conversely, transitioning from an active to an inactive lifestyle did not significantly alter the risk of mortality in either model.

For individuals with normal sleep duration (6–8 h), being active was associated with a significantly reduced risk of mortality in both models, with HR of 0.488 (95% CI 0.379–0.628) and 0.701 (95% CI 0.541–0.909) respectively. The transition from inactive to active was also associated with a significant reduction in mortality risk in the unadjusted model (HR 0.648, 95% CI 0.528–0.794), but this association was not significant in the adjusted model. Transitioning from an active to an inactive lifestyle showed a significant increase in mortality risk in the adjusted model (HR 1.342, 95% CI 1.074–1.677), indicating that maintaining an active lifestyle is beneficial.

Regarding those with long sleep duration (>8 h), the active lifestyle was associated with a significantly reduced risk of mortality in both models, with HR of 0.525 (95% CI 0.381–0.725) and 0.561 (95% CI 0.405–0.778) respectively. The transition from inactive to active was also associated with a significant reduction in mortality risk in both models, with HR of 0.721 (95% CI 0.595–0.875) and 0.785 (95% CI 0.646–0.955) respectively. Transitioning from an active to an inactive lifestyle did not significantly alter the risk of mortality.

The interaction terms were significant in the adjusted models for normal and long sleep durations, indicating that the effect of physical activity on mortality risk varies depending on sleep duration.

**Figure 1:**
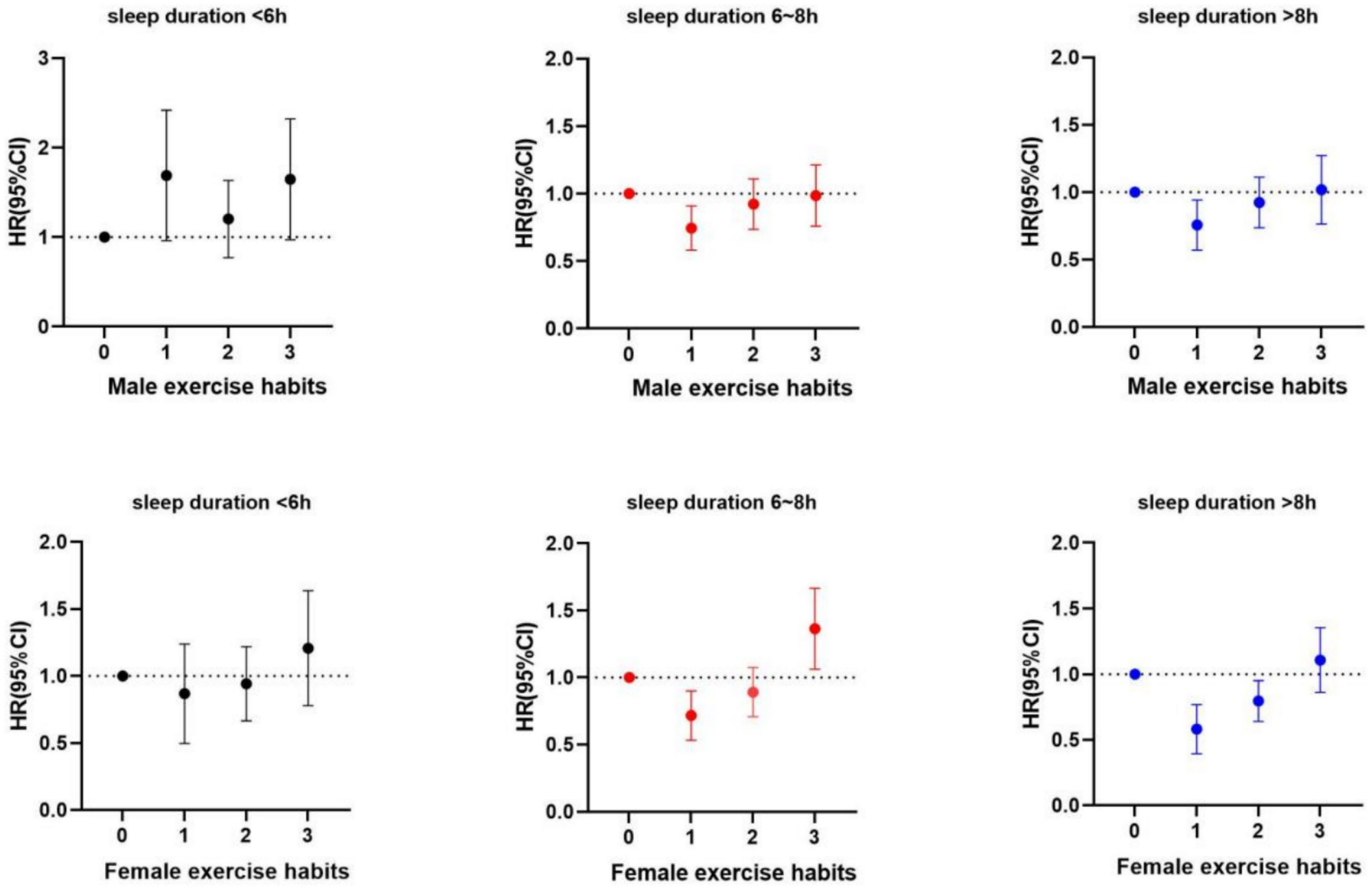
Forest plot of the joint effects of sleep duration and exercise habits all-cause mortality among older adult Chinese of different genders.

The results presented above are illustrated more clearly in Figure 1. For those with short sleep duration, the impact of exercise habits on mortality was not significant for either men or women. However, for individuals with normal and prolonged sleep, both males and females experienced a significant reduction in mortality by maintaining exercise habits. Additionally, older women who transitioned from a sedentary lifestyle to regular exercise during long sleep duration also exhibited lower mortality rates.

## 4 Discussion

The aim of this study was to investigate the joint effects of sleep duration and exercise habits on all-cause mortality in Chinese older adults. We analyzed the effects of sleep duration and exercise habits on mortality separately, and then further explored the joint effect of the two in order to gain a deeper understanding.

First, regarding the impact of sleep duration on mortality, we found that long sleep duration in older adults from China is significantly associated with an increased risk of mortality, aligning with existing literature that highlights the negative health implications of extreme sleep variations. Although adequate sleep is beneficial, prolonged sleep may indicate underlying health issues such as chronic diseases (20) and depression (21). Even after adjusting for covariates, the relationship between long sleep and increased mortality remained significant. Possible mechanisms include reduced physical activity (22), raising the risk of cardiovascular diseases (23, 24) and metabolic syndrome (25). Prolonged sleep correlates with chronic inflammation (26), endocrine disruption (27), and metabolic dysregulation (28), potentially leading to insulin resistance and increased risks of cardiovascular diseases and diabetes. Moreover, long sleep duration may relate to socioeconomic factors, mental health conditions, and lifestyle habits in older adults (29). Additionally, extended sleep can reduce social engagement, increasing feelings of loneliness and isolation (30). Our study also observed that among short sleepers, exercise habits did not significantly impact mortality for either sex. This finding is intriguing and warrants further exploration. One possible explanation is that short sleepers may already be at a higher baseline risk due to insufficient rest, and the protective effects of exercise may be overshadowed by the negative impacts of sleep deprivation (31). Another possibility is that the intensity or duration of exercise in this population may not be sufficient to counteract the adverse effects of short sleep (32). Alternatively, other lifestyle factors or comorbidities in short sleepers could interact with exercise habits in complex ways, masking any potential mortality benefits (32). Ultimately, understanding these complex interactions between sleep patterns and lifestyle factors is crucial for developing interventions to improve the health and well-being of older adults.

Secondly, studies of the impact of exercise habits on mortality in the older adult have found that maintaining exercise habits or switching from inactivity to exercise significantly reduced mortality among Chinese older adults. This is primarily because regular participation in physical activity improves physical function and mental health in older adults (33). For example, a study found that older adults who participated in a community-based exercise programmer showed significant improvements in physical functioning, ability to perform activities of daily living, and exercise self-efficacy (34). In addition, exercise has been strongly associated with mental health, with higher levels of moderate to vigorous physical activity associated with lower depressive symptoms (35). Among older adults, maintenance of exercise habits not only contributes to improved physical fitness but also enhances social support and self-efficacy, all of which are important factors in promoting healthy aging (36). Therefore, encouraging older adults to maintain an exercise habit or participate in exercise activities is an effective strategy to promote their health and longevity.

Finally, the joint effect of sleep duration and exercise habits on mortality in Chinese older adults was analyzed stratified by gender. The results indicated that for older Chinese men, maintaining a regular exercise routine seemed to eliminate the negative health effects associated with prolonged sleep. Specifically, the risk of death did not significantly increase even with extended sleep, provided that a consistent exercise regimen was followed. Notably, we also observed a significant interaction between normal sleep duration and exercise habits in older men. This suggests that those who maintain both normal sleep duration and regular exercise habits may experience the lowest risk of mortality. This is consistent with Duarte Junior’s findings that meeting moderate-to-vigorous physical activity recommendations reduces the risk associated with short or long sleep periods (4). The underlying mechanisms through which exercise mitigates the negative effects of prolonged sleep may involve both physiological and psychological pathways. Physiologically, regular physical activity can reduce systemic inflammation, a common pathway linking sleep duration and mortality (37). Exercise stimulates the release of anti-inflammatory cytokines, such as IL-6, which inhibit pro-inflammatory factors and improve overall health outcomes. Additionally, exercise can enhance cardiovascular and metabolic health, counteracting the adverse effects of prolonged sleep on these systems (38). Psychologically, physical activity can improve mental health by reducing depression and anxiety, both of which are associated with poor sleep quality and increased mortality (39). In contrast, while the overall trends for older women were similar to those of men, some unique observations emerged. Specifically, we found that women who experienced prolonged sleep had a significantly lower risk of death when they transitioned from inactivity to beginning an exercise routine. This indicates that, for this group of women, even with poor sleep patterns (such as long sleep duration), the risk of mortality can still be effectively mitigated by adopting new exercise habits. This finding suggests that health intervention strategies for older women should place special emphasis on fostering and maintaining exercise habits, particularly in the context of suboptimal sleep behaviors. Biologically, this may be explained by the fact that women tend to have a stronger response to the anti-inflammatory and metabolic benefits of exercise compared to men (40). Socioculturally, older women may face unique barriers to maintaining an active lifestyle, such as encountering greater challenges in initiating and sustaining regular exercise routines, and interventions targeting this group should focus on overcoming these barriers to promote sustained physical activity (41).

The findings of this study further support the critical roles of both exercise and sleep in promoting health among older adults (42). Additionally, the observed gender differences may reflect varying physiological and psychological responses to exercise and sleep. Therefore, gender-specific considerations should be taken into account when developing health coaching strategies for older adults to ensure that these strategies effectively address the unique health needs of different genders.

The study primarily relied on data gathered from questionnaires and objective data monitoring, which may introduce some bias. Therefore, future studies should validate these findings with larger sample sizes and more comprehensive data collection. It is also important to explore in greater depth the differences in the effects of exercise habits and sleep duration across various groups of older adults.

## 5 Conclusion

In conclusion, for older adults in China, both sleep duration and exercise habits are associated with all-cause mortality, and they also have combined effects on mortality risk. Significantly, the joint effects varies between males and females. Therefore, targeted health policies are essential to encourage this demographic to enhance their sleep and exercise habits. These policies should consider gender differences and provide individualized support and resources tailored to various groups. By implementing such measures, we can more effectively reduce mortality risk and improve the overall quality of life for older individuals.

## Data Availability

The raw data supporting the conclusions of this article will be made available by the authors, without undue reservation.

## Footnotes

^1^ https://www.medsta.cn/software
