# Supplementary material for: The joint effects of sleep duration and exercise habit on all-cause mortality among Chinese older adult: a national community-based cohort study
Source: Front Public Health. 2025 Mar 26;13:1538513. doi: 10.3389/fpubh.2025.1538513 (PMC11978651; doi:10.3389/fpubh.2025.1538513)
Supplement: Supplementary file 1 [file Data_Sheet_1.PDF]

Supplementary material 1

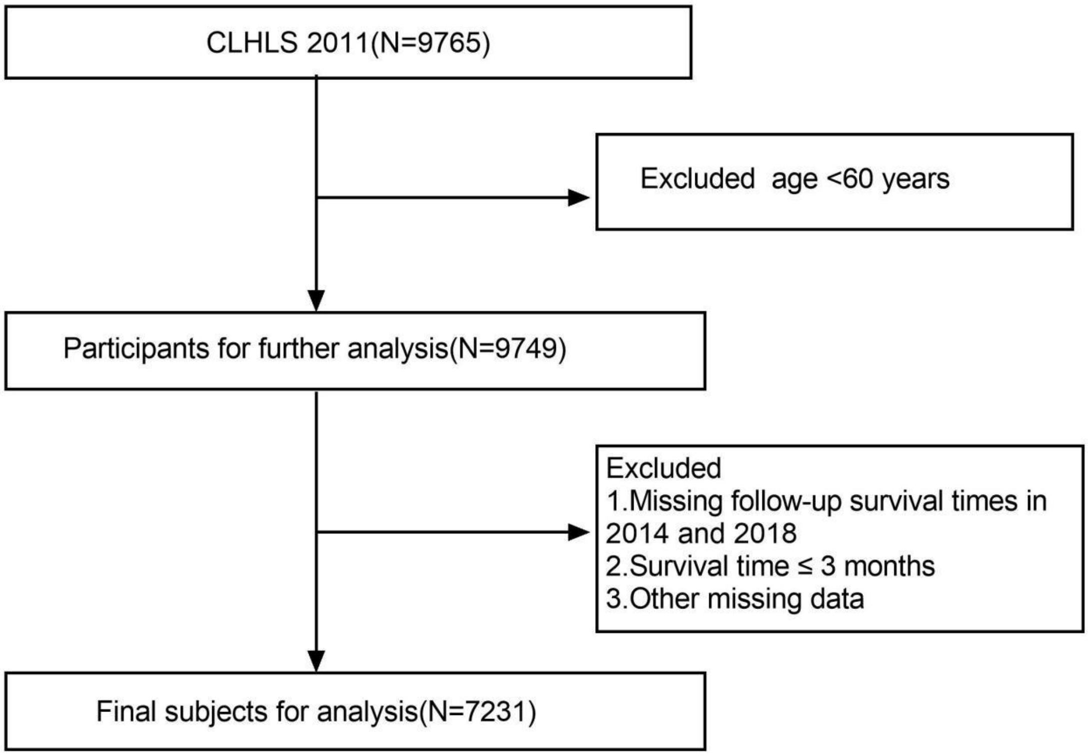

SFig. 1 Flow chart of this analysis based on CLHLS 2011-2018

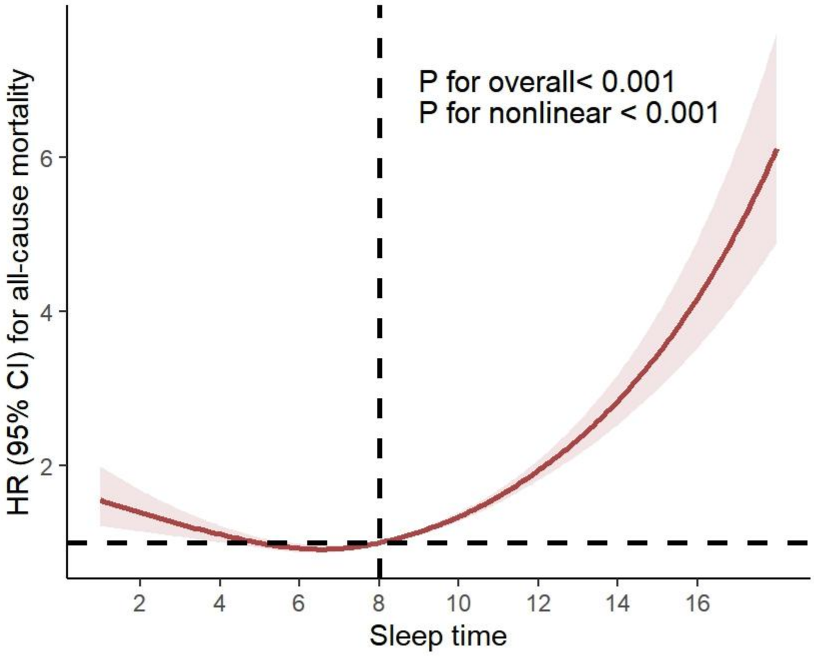

*S*Fig. 2 Restricted cubic spline curves for the relationship between sleep time and all-cause mortality in Chinese older adults

Supplementary material 2

Using the Martingale residual and Schoenfeld residual method to test the proportional hazards

1．Martingale residual analysis：The Martingale residual plot（*S*Fig. 3） indicates that the model has a good fitting effect, with residuals randomly distributed around 0. Although some residual points deviate from the main distribution area, the majority of residual points are concentrated around 0 and there is no obvious systematic bias or trend. Therefore, these outliers may be within an acceptable range.

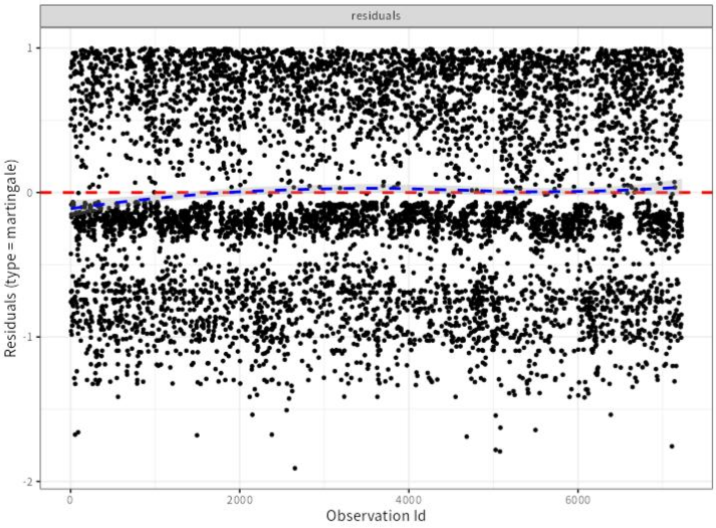

*S*Fig. 3 The Martingale residual plot

2．Schoenfeld residual analysis：

1）Generate Schoenfeld residuals for each covariate.

2）Generate a time rank variable.

3）Conduct a correlation analysis between the residuals and the time rank.（see STab. 1）

STab.1 Analysis of the correlation between residuals of control variables and time rank

control variable	Pearson Correlation	*P*
Age	-0.014	0.060
Gender	-0.026	0.146
Marital status	0.029	0.109
Education	-0.011	0.530
Smoke	0.026	0.071
Drink	0.010	0.565
Economic state	0.033	0.067
Disease	-0.005	0.762

Health	0.022	0.219
BMI	0.002	0.979
sleep quality	0.019	0.283

The results in STab. 1 indicate that there is no significant linear relationship between the Schoenfeld residuals and the time ranks, suggesting that the control variables satisfy the proportional hazards assumption of the Cox proportional hazards model.
